# Exploring the bone sparing effects of postbiotics in the post-menopausal rat model

**DOI:** 10.1186/s12906-021-03327-w

**Published:** 2021-05-28

**Authors:** Nima Montazeri-Najafabady, Younes Ghasemi, Mohammad Hossein Dabbaghmanesh, Yousef Ashoori, Pedram Talezadeh, Farhad Koohpeyma, Seyedeh Narjes Abootalebi, Ahmad Gholami

**Affiliations:** 1 Endocrinology and Metabolism Research Center, Shiraz University of Medical Sciences, Shiraz, Iran; 2 Biotechnology Research Center, Shiraz University of Medical Sciences, P.O. Box: 71348-14336, Shiraz, Iran; 3 Pharmaceutical Science Research Center, Shiraz University of Medical Sciences, Shiraz, Iran; 4 Division of Intensive Care Unit, Department of Pediatrics, School of Medicine, Shiraz University of Medical Sciences, Shiraz, Iran

**Keywords:** Postbiotics, Probiotics lysates, Post-menopausal osteoporosis, Ovariectomized rat, Bioactive compounds

## Abstract

**Background:**

Post-menopausal osteoporosis is a concern of health organizations, and current treatments do not seem enough. Postbiotics as bioactive compounds produced by probiotics may be an attractive alternative for bone health. In this study, we prepared, formulated, and compared the effects of cell lysate and supernatant of five native probiotic strains (*Lactobacillus acidophilus*, *Lactobacillus reuteri*, *Lactobacillus casei*, *Bifidobacterium longum*, and *Bacillus coagulans*) in ovariectomized (OVX) rats.

**Methods:**

The probiotic strains were isolated, and their cell-free supernatants and biomasses as postbiotics were extracted and formulated using standard microbial processes. The Sprague-Dawley rats were fed by 1 × 10^9^ CFU/ml/day postbiotic preparations for 4 weeks immediately after ovariectomy. Dual-energy X-ray absorptiometry (DEXA) scans were accomplished to evaluate femur, spine, and tibia BMD. The serum biochemical markers [calcium, phosphorus, and alkaline phosphatase] were assessed.

**Results:**

Postbiotics could considerably improve the global and femur area in OVX rats. In the case of global bone mineral density (BMD), *Lactobacillus casei* lysate and supernatant, *Bacillus coagulans* lysate and supernatant, lysate of *Bifidobacterium longum* and *Lactobacillus acidophilus*, and *Lactobacillus reuteri* supernatant significantly increased BMD. We found *Bacillus coagulans* supernatant meaningfully enriched tibia BMD.

**Conclusion:**

Postbiotic could ameliorate bone loss resulting from estrogen deficiency. Also, the effects of postbiotics on different bone sites are strain-dependent. More clinical studies need to explore the optimal administrative dose and duration of the specific postbiotics in protecting bone loss.

## Background

As a primary concern of health organizations, osteoporosis is described by an imbalance between osteoblast and osteoclast activity, which leads to conceded bone strength and elevation of fracture risk [1, 2]. Aging is one of the leading causes of osteoporosis and is directly linked to its prevalence [3]. Post-menopausal women and older adults are more susceptible than other population groups to develop osteoporosis [4]. Currently, the existing treatment for post-menopausal osteoporosis is including estrogen replacement and perhaps calcitonin, along with calcium and vitamin D supplementation, selective estrogen-receptor modulator (SERM), bisphosphonates, a human monoclonal antibody to the receptor activator of nuclear factor-κB (NF-κB) ligand (RANKL; denosumab), and the parathyroid hormone analog teriparatide [5]. However, these medications have not been sufficient for osteoporosis treatment and are associated with many adverse effects such as upper gastrointestinal discomfort, venous thromboembolism, and increased cancer rates [5]. So that finding alternative treatment with negligible side effects is urgently needed. Since gut microbiota (GM) alteration influences bone homeostasis, it is logical to change the microbiota to induce beneficial skeletal effects. Microbiota manipulation via supplementation with probiotics is an excellent strategy to prevent bone loss [6]. As reported previously, sex steroid deficiency associated-bone loss is microbiota dependent and prevented by probiotics [7].

Probiotics are live microorganisms that are suitable for the treatment of various diseases [8–12]. According to the Food and Agriculture Organization (FAO) and the World Health Organization (WHO), which was followed by the International Scientific Association for Probiotics and Prebiotics (ISAPP), probiotics are live strains of microorganisms that confer health benefits upon the host when administrated in adequate amounts [13].

Various types of probiotics, especially *Lactobacillus* spp. and *Bifidobacterium* spp., have been reported to modify the composition of GM and elicit positive effects on bone in both healthy and pathologic conditions [14–16]. Chiang and Pan reported using *Lactobacillus paracasei* (NTU 101) and *Lactobacillus plantarum* (NTU 102) (1 × 10^8^ CFU/mL) in ovariectomized (OVX) mice increased the bone trabecular number [17]. Kim et al. displayed that a reduced BMD level in OVX rats will be significantly ameliorated by administrating *Lactobacillus casei* fermented milk [18]. *Bifidobacterium longum* from fermented broccoli also showed a significant effect on bone health [19]. In another study, Ohlsson et al. found that treating mice with either the single *Lactobacillus* (L) strain, the *Lactobacillus paracasei* DSM13434, or a mixture of three strains, *L. paracasei* DSM13434, *L. plantarum* DSM 15312, and DSM 15313 protected mice from OVX-induced cortical bone loss and bone resorption [20]. In our recent study, we used native probiotic strains containing three *Lactobacillus* strains (*Lactobacillus acidophilus*, *Lactobacillus reuteri*, *Lactobacillus casei*), one *bifidobacterium* strain (*Bifidobacterium longum*), and one *Bacillus* strain (*Bacillus coagulans*) in OVX rats. We found that all probiotics ameliorated bone loss [21]. Although probiotics are known as safe for disease therapy, the administration of live organisms may result in severe infections and represent considerable risk, especially in severely ill patients [22]. There is growing evidence that comparable advantageous effects could be achieved with sterile lysates or components secreted from probiotics or even commensal microbes (postbiotics) [23].

Postbiotics are functional bioactive compounds produced by probiotics during the fermentation process, including metabolites, short-chain fatty acids (SCFAs), microbial cell fractions, functional proteins, extracellular polysaccharides (EPS), cell lysates, teichoic acid, peptidoglycan-derived muropeptides, and pili-type structures [12, 24]. Many of the proposed health effects of the addition of probiotics are related to the cell lysate and supernatants [25]. It has been suggested that using postbiotics could be an attractive alternative for other’-biotics’ in critically ill patients, young children, and premature neonates [26, 27].

To understand the positive effects of postbiotics on bone health, we prepared, formulated, and compared cell lysate and supernatant of five native probiotic strains (*Lactobacillus acidophilus*, *Lactobacillus reuteri*, *Lactobacillus casei*, *Bifidobacterium longum*, and *Bacillus coagulans*) in OVX rats. According to our best knowledge, this is the first study examining the effects of postbiotics on bone health and homeostasis.

## Material and methods

### Probiotic strains

The probiotics, including *Lactobacillus (L.) acidophilus*, *L. casei*, *L. reuteri, Bifidobacterium longum,* and *Bacillus coagulans,* were isolated from fermented dairy products, which have been characterized in our previous studies [8, 21]. Biochemical, molecular, and other features of strains were previously described [28].

### Postbiotic preparation and formulation

All probiotic strains were cultured in 300 mL De Man, Rogosa, and Sharpe (MRS) broth at 37 °C in a microaerophilic jar for 24 h to reach the bacterial concentration of 1 × 10^9^ CFU/mL. Then, the cultures were centrifugated at 10000×g for 15 min at 4 °C. The cell-free supernatants were collected and ultra-filtrated through a 0.22 μm cellulose acetate membrane (Sartorius, Göttingen, Germany). The supernatants were used for a biological evaluation immediately after preparation.

The biomasses were also washed three times with sterile phosphate-buffered saline (PBS) and treated with a French press. Then, the probiotic biomasses were freeze-dried and stored at − 20 °C until used for animal studies. Before biological evaluations, the biomasses were diluted to a concentration of 30 g/L. For the final formulation, the bacterial lysates were heated at 60 °C for 30 min, dissolved in PBS (pH 7.4), and were shaking for 30 min.

Both postbiotic groups (supernatants and bacterial lysates) were tested for sterility aerobically and anaerobically by cultivation for 48 h.

### Animals and intervention procedure

Eighty-four Sprague-Dawley rats aged 12–14 weeks (weight 200 ± 20 g) and females were purchased from the Laboratory Animal Center of Shiraz University of Medical Sciences. The rats were housed under particular pathogen-free conditions (room temperature with the relative humidity of 60 ± 5%, the temperature of 23 ± 2 °C, and 12/12 h light/dark cycles) at the animal lab of endocrinology and metabolism research center and administered pellet diet (rodent chow; Behparvar Co., Tehran, Iran) and water ad libitum. Rodent chow compositions are as follows: Crude protein 23%, crude fat 3.5%, crude fiber 4.5%, ash 10%, calcium 0.95–1%, phosphorus 0.65–0.7%, NaCl 0.5%, lysine 1.15%, methionine 0.33%, threonine 0.72%, tryptophan 0.25%, cysteine 0.3%). Three months after ovariectomy, treatments were initiated. Postbiotics were delivered to animals by gavage. The intervention period was 4 weeks. At the end of the treatment period, rats were sacrificed and used for DEXA analysis [21, 29]. One week after rats’ adaption to the animal room, they were allocated to twelve groups (seven rats in each group): (1) Control group: fed with normal saline; (2) OVX group: fed with normal saline; (3) OVX + *Lactobacillus acidophilus* supernatant: fed with 1 × 10^9^ CFU/ml/day of *Lactobacillus acidophilus* supernatant; (4) OVX + *Lactobacillus casei* supernatant: fed with 1 × 10^9^ CFU/ml/day of *Lactobacillus casei* supernatant; (5) OVX + *Lactobacillus reuteri* supernatant: fed with 1 × 10^9^ CFU/ml/day of *Lactobacillus reuteri* supernatant; (6) OVX + *Bacillus coagulans* supernatant: fed with 1 × 10^9^ CFU/ml/day of *Bacillus coagulans* supernatant; (7) OVX + *Bifidobacterium longum* supernatant: fed with 1 × 10^9^ CFU/ml/day of *Bifidobacterium longum* supernatant; (8) OVX + *Lactobacillus acidophilus* lysate: fed with 1 × 10^9^ CFU/ml/day of *Lactobacillus acidophilus* lysate; (9) OVX + *Lactobacillus casei* lysate: fed with 1 × 10^9^ CFU/ml/day of *Lactobacillus casei* lysate; (10) OVX + *Lactobacillus reuteri* lysate: fed with 1 × 10^9^ CFU/ml/day of *Lactobacillus reuteri* lysate; (11) OVX + *Bacillus coagulans* lysate: fed with 1 × 10^9^ CFU/ml/day of *Bacillus coagulans* lysate; (12) OVX + *Bifidobacterium longum* lysate: fed with 1 × 10^9^ CFU/ml/day of *Bifidobacterium longum* lysate. Postbiotics were delivered to animals by gavage. The intervention period was 4 weeks. At the end of the treatment period, rats were sacrificed and used for DEXA analysis. This work was approved by the Ethics Committee of Shiraz University of Medical Sciences, Shiraz, Iran, under ID code IR.SUMS.REC. 97–01–33-18,580.

### Ovariectomy

The adult female rats were ovariectomized bilaterally under anesthesia by ketamine 10% (100 mg/kg, Alfasan, the Netherlands) and xylazine 2% (10 mg/kg, Alfasan, the Netherlands). Both ovaries were surgically removed, except for the control group, after joining the uterine horn through a midline longitudinal incision. Postoperatively, morphine injection of (5 mg/kg S.C) was used to reduce pain in rats, and oxytetracycline spray was used to prevent infection.

### Dual-energy X-ray absorptiometry parameter measurements

Dual-energy X-ray absorptiometry (DEXA) scans were accomplished on a Discovery QDR, USA device using the specific software for small animals to evaluate femur, spine, and tibia bone mineral density (BMD, g/cm^2^) at the end of the experiment. DEXA was performed after the animals were sacrificed. All the rats were euthanized with ketamine and xylazine solution intraperitoneally according to the AVMA Guidelines for the euthanasia of animals and sacrificed using thiopental (100 mg/kg) at the experiment termination. At first, we set up the RAT STEP PHANTOM (Hologic P/N010-0758Rev.004) scan. In this method, when the system motion was completed, we centered the STEP PHANTOM on the table along the long axis of the laser with the cross-hair ¾ ^“(^2 cm) of the right edge of the thinnest step. Then we pressed a continue button to start the scan. The protocols of the study were approved by the Institutional Animal Ethics Committee of Shiraz University of Medical Sciences (Shiraz, Iran), following NIH guidelines for the care and use of animals (NIH publication No. 85–23, revised in 1996).

### Assay for biochemical markers

Blood samples were collected in chilled non-heparinized tubes to clot at room temperature by cardiocentesis. The blood samples were centrifuged (3500 rpm at 4 °C for 20 min), and the separated sera were used for biochemical analysis. A spectrophotometric device (BT 1500 Auto-analyzer) was used to evaluate the serum biochemical markers [Calcium (Ca), Phosphorus (P), and Alkaline phosphatase (ALP)].

### Statistical analysis

The data are revealed as the mean ± standard deviation (SD). IBM© SPSS© Statistics v 22.0 package for Windows was applied to perform statistical analyses. One-way ANOVA was conducted to explore the differences of biochemical parameters (Calcium, Phosphorus, and Alkaline phosphatase) and bone densitometry parameters [BMD, bone mineral content (BMC), and Area] between groups. Graphs were drawn using GraphPad Prism 5 (GraphPad Inc., La Jolla, CA, USA). Tukey post hoc analysis was executed when the outcomes of ANOVA indicated significance (*P* ≤ 0.05).

## Results

### Changes in calcium, phosphorus, and alkaline phosphatase after supplementation with postbiotics

**Fig. 1:**
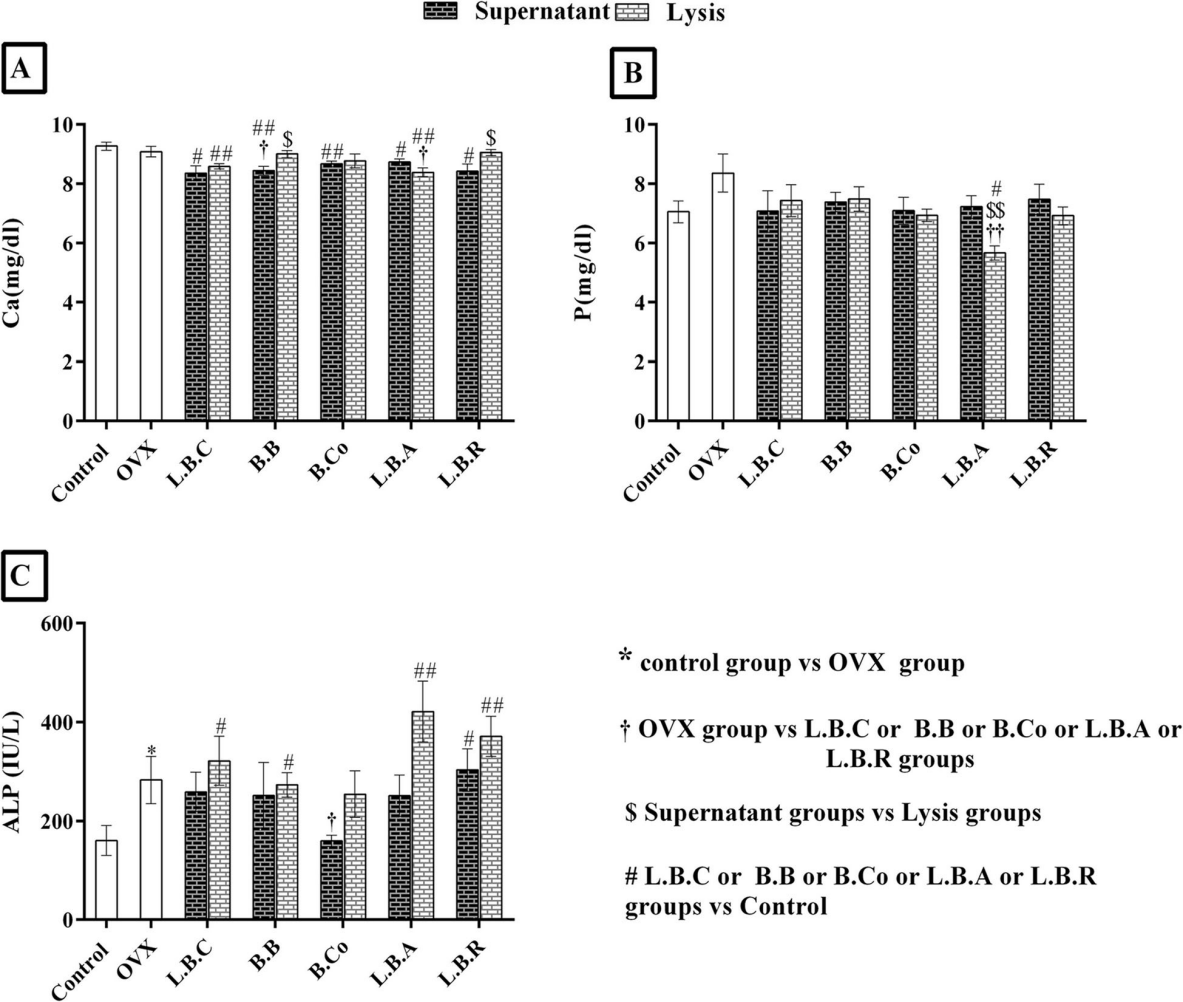
The effect of postbiotics (supernatant and bacterial lysate) on Ca (**a**), P (**b**) and ALP (**c**) of ovariectomized rats, 4 weeks after treatment. All differences were considered significant at *P* < 0.05. †: OVX group vs probiotic groups; † (*P* < 0.05), †† (*P* < 0.01), ††† (*P* < 0.001). $: Supernatant groups vs Lysate groups; $ (*P* < 0.05), $$ (*P* < 0.01). #: Probiotic groups vs Control group; # (*P* < 0.05), ## (*P* < 0.01): Probiotic groups vs Control. OVX: ovariectomized rats, LBC: *Lactobacillus casei*, BB: *Bifidobacterium longum*, BCO: *Bacillus coagulans*, LBA: *Lactobacillus acidophilus,* LBR: *Lactobacillus reuteri*

The results demonstrated that calcium (Ca) concentration in serum was lower in the OVX group compared to the control group at the end of the experiment, but the difference was not significant. In all postbiotic treated groups except the group treated with *Lactobacillus acidophilus*, the Ca concentration was higher in lysate groups in comparison with supernatant groups (Fig. 1a). These differences were significant (*P* ≤ 0.05) only in groups supplemented with *Lactobacillus reuteri* and *Bifidobacterium longum*. The concentration of Ca was significantly (P ≤ 0.05) lower in all groups compared to the control group except OVX + *Lactobacillus reuteri* lysate, OVX + *Bifidobacterium longum* lysate, and OVX + *Bacillus coagulans* lysate groups (Fig. 1a).

As expected, the serum P concentration was higher in the OVX group vs. the control group. Supplementation with postbiotic s decreased P concentration in all groups, but this difference was only significant (*P* ≤ 0.05) in the OVX + *Lactobacillus acidophilus* lysate group compared to both control and OVX groups (Fig. 1b).

As the results showed, ALP concentration was significantly (*P* ≤ 0.05) more significant in the OVX group vs. the control group. Treatment with *Bacillus coagulans* supernatant considerably reduced ALP concentration. In other groups, ALP concentrations were elevated after treatment with postbiotic compared to the control group. We observed that ALP concentrations were meaningfully (*P* ≤ 0.05) higher for each postbiotic separately in lysates groups versus supernatant groups (Fig. 1c).

### Global, spine, and femur area of OVX rats were ameliorated after supplementation with postbiotics

**Fig. 2:**
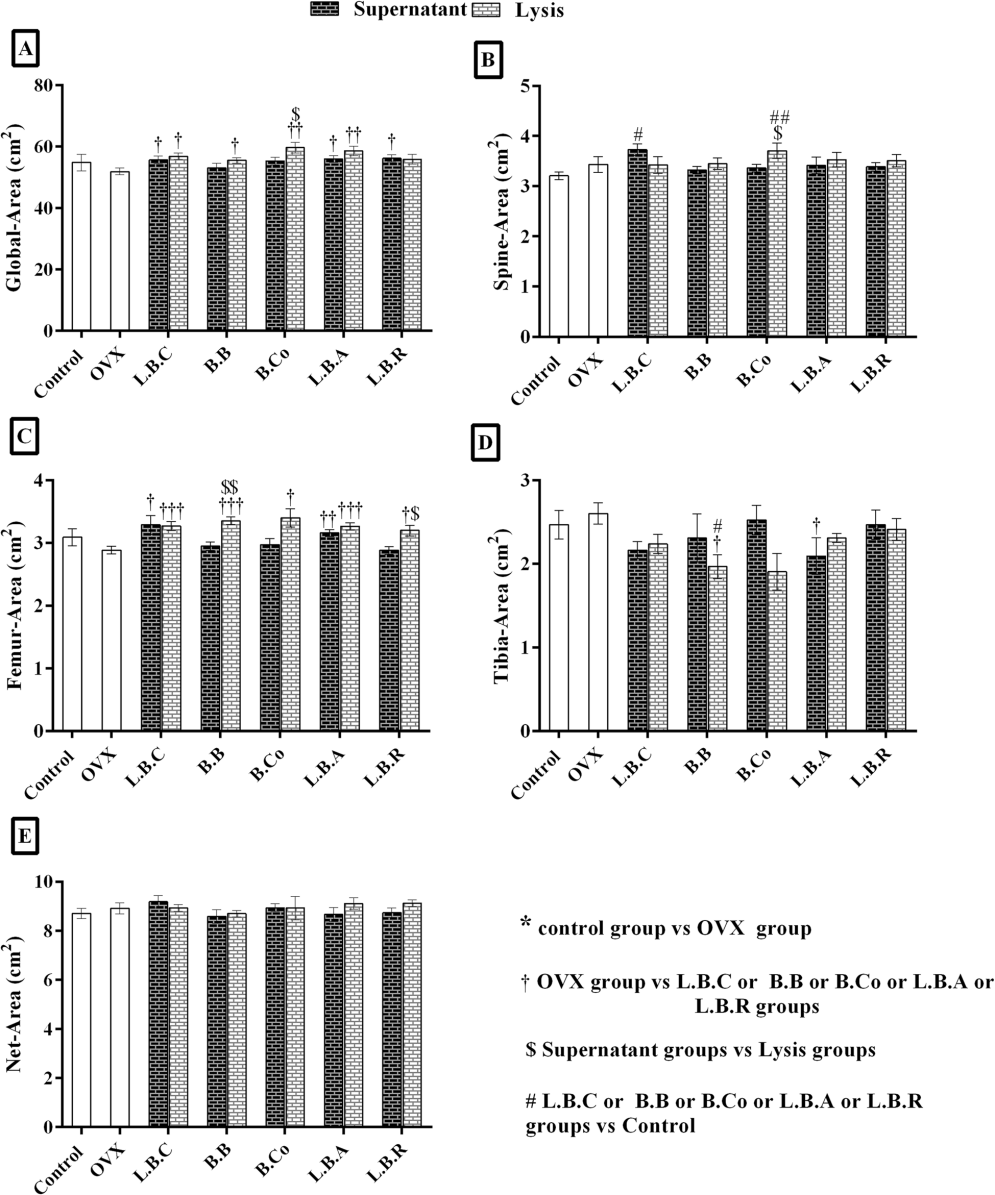
The effect of postbiotics (supernatant and bacterial lysate) on the global area (**a**), spine area (**b**), femur area (**c**) and tibia area (**d**) of ovariectomized rats, 4 weeks after treatment. All differences were considered significant at *P* < 0.05. †: OVX group vs probiotic groups; † (*P* < 0.05), †† (*P* < 0.01), ††† (*P* < 0.001). $: Supernatant groups vs Lysate groups; $ (*P* < 0.05), $$ (*P* < 0.01). #: Probiotic groups vs Control group; # (*P* < 0.05), ## (*P* < 0.01): Probiotic groups vs Control. OVX: ovariectomized rats, LBC: *Lactobacillus casei*, BB: *Bifidobacterium longum*, BCO: *Bacillus coagulans*, LBA: *Lactobacillus acidophilus,* LBR: *Lactobacillus reuteri*

The global area was significantly (*P* ≤ 0.05) enhanced in all postbiotic-treated groups compared to the OVX group except for OVX *+ Bifidobacterium longum* supernatant, OVX + *Bacillus coagulans* supernatant, and OVX + *Lactobacillus reuteri* lysate (Fig. 2a). *Lactobacillus casei* supernatant and *Bacillus coagulans* lysate significantly (P ≤ 0.05) increased spine area in OVX rats compared to the control group. No significant differences were detected in other groups (Fig. 2b). The femur area was considerably higher in OVX + *Lactobacillus casei* supernatant, OVX + *Lactobacillus casei* lysate, OVX *+ Bifidobacterium longum* lysate, OVX + *Bacillus coagulans* lysate, OVX + *Lactobacillus acidophilus* lysate, OVX + *Lactobacillus acidophilus* supernatant, OVX + *Lactobacillus reuteri* lysate groups compared to OVX group (Fig. 2c). No significant variation was observed between OVX *+ Bifidobacterium longum* supernatant, OVX + *Bacillus coagulans* supernatant, OVX + *Lactobacillus reuteri* supernatant, and OVX group. In contrast to the global, spine, and femur area, the tibia area was lesser in all of the postbiotics-treated group versus the OVX group (Fig. 2d). The differences were significant for *Bifidobacterium longum* lysate and *Lactobacillus acidophilus* supernatant.

### Postbiotics supplementation improve global, spine, femur, and tibia BMC in OVX rats

**Fig. 3:**
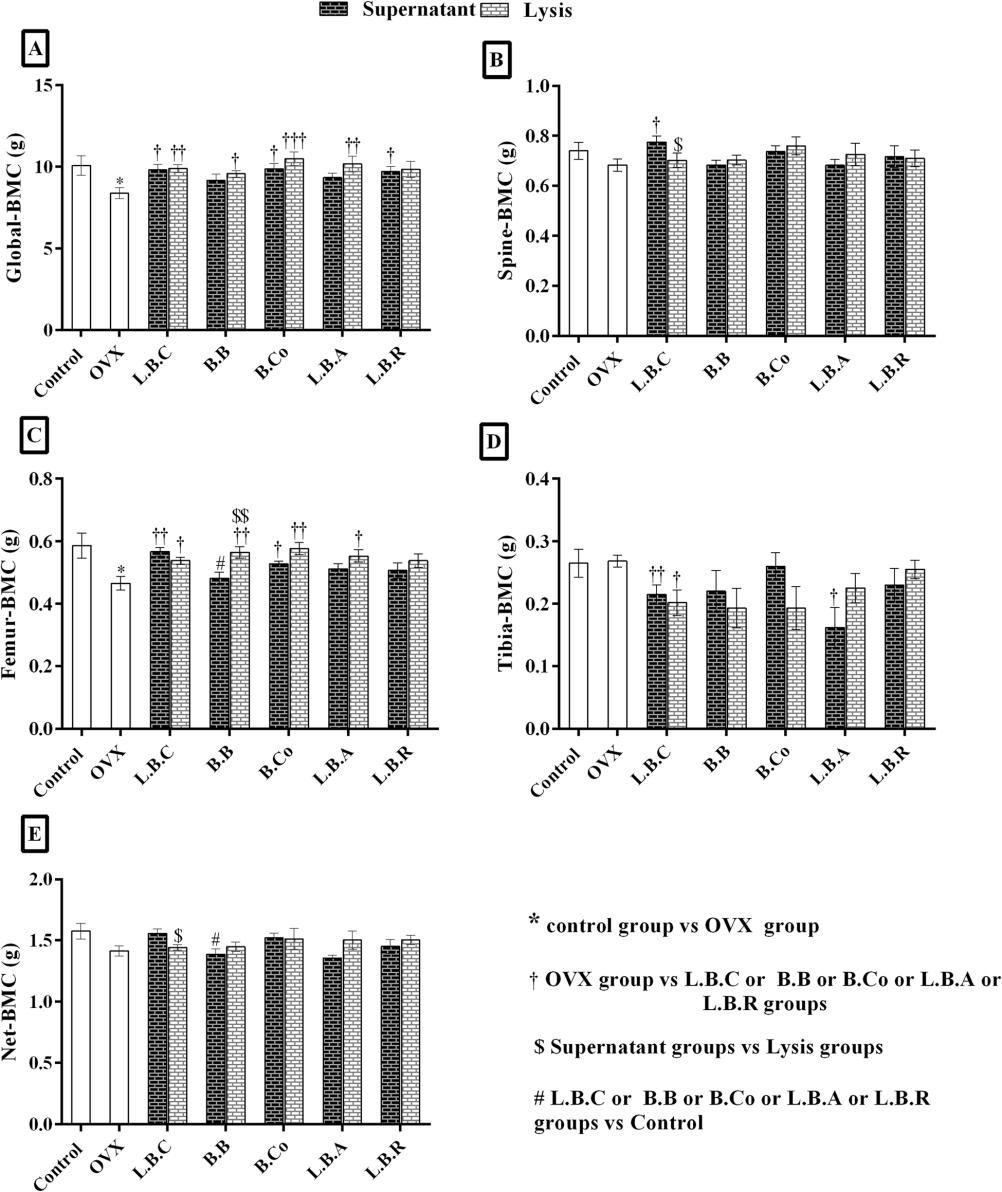
The effect of postbiotics (supernatant and bacterial lysate) on the bone mineral content (BMC) of global (**a**), spine (**b**), femur (**c**) and tibia (**d**) of ovariectomized rats, 4 weeks after treatment. All differences were considered significant at *P* < 0.05. *: Control group vs OVX group; * (*P* < 0.05). †: OVX group vs probiotic groups; † (*P* < 0.05), †† (*P* < 0.01), ††† (*P* < 0.001). $: Supernatant groups vs Lysate groups; $ (*P* < 0.05), $$ (*P* < 0.01). #: Probiotic groups vs Control group; # (*P* < 0.05), ## (*P* < 0.01): Probiotic groups vs Control. OVX: ovariectomized rats, LBC: *Lactobacillus casei*, BB: *Bifidobacterium longum*, BCO: *Bacillus coagulans*, LBA: *Lactobacillus acidophilus,* LBR: *Lactobacillus reuteri*

Global, spine, and femur (BMC) decreased in the OVX group compared to the control group, but it was not significant (Fig. 3). Postbiotics significantly (*P* ≤ 0.05) improved Global BMC in the OVX group except for OVX *+ Bifidobacterium longum* supernatant, OVX + *Lactobacillus acidophilus* supernatant, and OVX + *Lactobacillus reuteri* lysate groups (Fig. 3a). Spine BMC has significantly enhanced only in OVX + *Lactobacillus casei* supernatant and OVX + *Lactobacillus casei* lysate groups (Fig. 3b). Femur BMC increased dramatically after supplementation with postbiotics except for *Bifidobacterium longum* supernatant, *Lactobacillus acidophilus* supernatant, and *Lactobacillus reuteri* lysate, and supernatant (Fig. 3c). There was a significant difference between OVX *+ Bifidobacterium longum* supernatant and OVX *+ Bifidobacterium longum* lysate groups. Tibia BMC was significantly (*P* ≤ 0.05) lower in OVX groups treated with *Lactobacillus casei* (lysate and supernatant) and *Lactobacillus acidophilus* supernatant, but no significant differences were observed in other OVX groups treated with postbiotics (Fig. 3d). Spine and net BMC were significantly (P ≤ 0.05) different between *Lactobacillus casei* lysate and supernatant.

### *Bacillus coagulans* supernatant improved global and spine BMD in OVX rats

**Fig. 4:**
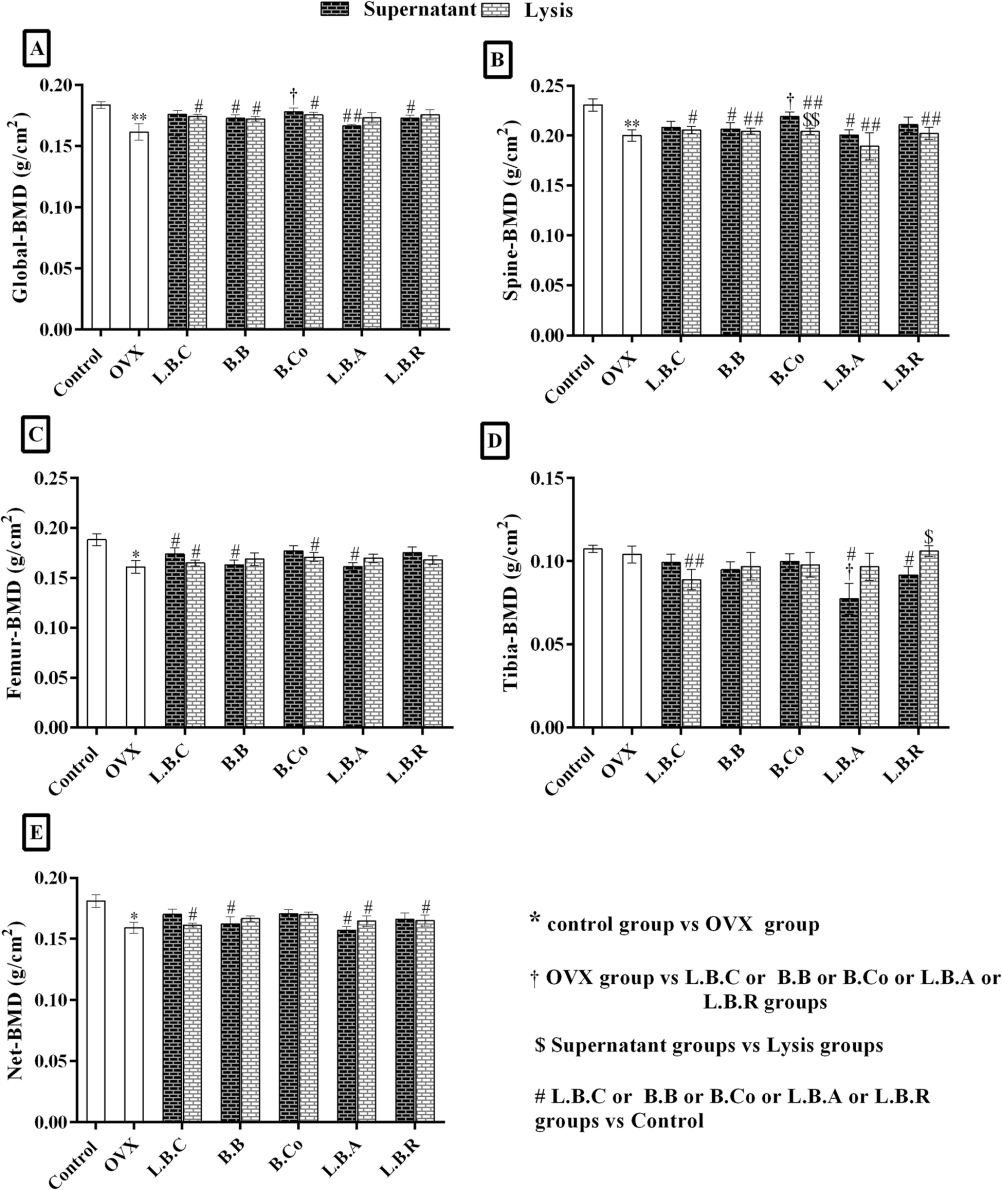
The effect of postbiotics (supernatant and bacterial lysate) on the bone mineral density (BMD) of global (**a**), spine (**b**), femur (**c**) and tibia (**d**) of ovariectomized rats, 4 weeks after treatment. All differences were considered significant at *P* < 0.05. *: Control group vs OVX group; * (*P* < 0.05), ** (*P* < 0.01). †: OVX group vs probiotic groups; † (*P* < 0.05), †† (*P* < 0.01), ††† (*P* < 0.001). $: Supernatant groups vs Lysate groups; $ (*P* < 0.05), $$ (*P* < 0.01). #: Probiotic groups vs Control group; # (*P* < 0.05), ## (*P* < 0.01): Probiotic groups vs Control. OVX: ovariectomized rats, LBC: *Lactobacillus casei*, BB: *Bifidobacterium longum*, BCO: *Bacillus coagulans*, LBA: *Lactobacillus acidophilus*, LBR: *Lactobacillus reuteri*

Global BMD increased after supplementation with postbiotics, but it was only significant (*P* ≤ 0.05) for *Bacillus coagulans* supernatant (Fig. 4a). In terms of spine BMD, none of the postbiotics improved spine BMD except for *Bacillus coagulans* supernatant compared to the untreated OVX group (according to the graphic shown in Fig. 4b). *Bacillus coagulans* supernatant significantly enhanced spine BMD compared to the untreated OVX group (Fig. 4b). No significant differences were observed for femur BMD after administering postbiotics cell lysate and supernatant to OVX rats (Fig. 4c). Regarding tibia BMD, OVX did not cause significant bone loss animal tibia. Moreover, in postbiotic-treated rats, tibia BMD is not significantly different from the OVX untreated group (Fig. 4d).

## Discussion

The latest investigations indicated a close relation between gut microbiota composition and bone homeostasis [30] and probiotics in gut-bone signaling [14]. Our previous study revealed the supportive role of probiotic live cells in protecting rats from ovariectomy-induced bone loss [21]. This study is a novel in which we explored the effects of postbiotics (lysate and supernatant of probiotics) in protecting rats from bone loss induced by ovariectomy. The impacts of postbiotics on various bone compartments have been investigated in the current study. The results showed that postbiotics could not significantly increase serum Ca concentration compared to the OVX untreated group.

In contrast, Ghanem et al. reported that probiotic yogurt enriched with *L. reuteri* enhanced calcium absorption in growing rats [31]. Perez-Conesa et al. explained that *Bifidobacterium bifidum* and *Bifidobacterium longum* augmented apparent absorption and apparent calcium retention in weanling rats [32]. Yan et al. indicated that dietary supplementation of a *Bacillus subtilis* based probiotic improves broiler bone traits, most likely through increased calcium intestinal absorption and reduced bone resorption by inhibiting sympathetic activity via the central serotonergic system [33]. A study on *Lactobacillus casei*, *Lactobacillus reuteri*, and *Lactobacillus gasseri* reported higher apparent calcium absorption in growing rats and 35% higher bone weight than the control group [31].

In line with our recent study, *Lactobacillus acidophilus* lysate significantly decreased serum phosphorus compared to the untreated OVX group. Also, we found that *Bacillus coagulans* supernatant significantly decreased ALP compared to the OVX group, while no significant changes were detected for other postbiotic groups.

In the current study, we also investigated the effects of postbiotics (lysate and supernatant of probiotics) on bone quality (Area, BMC, and BMD). The results revealed that not only live probiotics but postbiotics could considerably improve the global and femur area in OVX rats. In agreement with our previous study, all postbiotics ameliorated the global area. For the femur area, *Lactobacillus casei* lysate and supernatant, *Bifidobacterium longum* lysate, *Bacillus coagulans* lysate, *Lactobacillus acidophilus* lysate, and supernatant, *Lactobacillus reuteri* lysate displayed positive effects. No significant improvement had been detected in the spine area in this study.

In contrast to our previous outcomes, *Bacillus coagulans* lysate and *Lactobacillus reuteri* supernatant decreased the tibia area, while similar to our previous study, no significant differences were found in other postbiotic supplemented groups. In the case of global BMC, *Lactobacillus casei* lysate and supernatant, *Bacillus coagulans* lysate and supernatant, lysate of *Bifidobacterium longum,* and *Lactobacillus acidophilus*, and *Lactobacillus reuteri* supernatant significantly increased BMC compared to the OVX group. In comparison with our previous study, *Lactobacillus casei*, *Lactobacillus acidophilus*, and *Lactobacillus reuteri* revealed similar results. Regarding spine BMC, only *Lactobacillus casei* supernatant considerably increased BMC, while our previous study on live probiotics showed no progressive effect on spine BMC. *Lactobacillus casei* and *Bacillus coagulans* lysate and supernatant, lysate of *Bifidobacterium longum,* and *Lactobacillus acidophilus* significantly enhanced femur BMC whereas previously, no significant differences were observed.

Perez-Conesa et al. recommended that increasing calcium absorption in the distal colon is directly associated with increasing calcium contents of the femur and tibia [32]. In agreement with Perez-Conesa et al. study, in the current study, we observed that OVX groups in which postbiotics increased serum calcium concentration (*Bifidobacterium longum* and *Bacillus coagulans*-treated groups) had higher femur BMC. We found that tibia BMC in *the Bacillus coagulans supernatant treated group was the same as in control and OVX untreated groups*. In the case of tibia BMC, similar results were detected for *Lactobacillus casei* compared to our previous work. *Bacillus coagulans* supernatant also increased global and spine BMD, while other postbiotics did not show significant changes. Lysate and supernatant of investigated strains like their live forms revealed no positive impact on femur BMD. *Lactobacillus casei* lysate and supernatant comparable to its live form increased tibia BMD. Similarly, Kim et al. specified that a decreased level of BMD in OVX rats would be significantly improved by administrating *Lactobacillus casei* 393 from fermented milk [18]. Bone formation and osteoblastic activity are described by serum ALP concentration [34]. There is clear evidence showing elevation of serum bone turnover markers such as ALP directly related to bone loss [35]. As expected, in the current study, OVX rats had elevated ALP levels compared to the control group. There was no significant difference between ALP concentration in OVX rats treated with postbiotics and untreated OVX group except for *Bacillus coagulans* supernatant. *Bacillus coagulans* supernatant treated group significantly decreased ALP concentration compared to untreated OVX group. These events may be primarily due to the effect of probiotic bacteria on the secretion pattern of parathyroid hormone and calcitonin. It was indicated that probiotic short-chain fatty acids reduce parathyroid hormone (PTH), increasing mineral absorption and decreasing ALP [36].

*Bacillus coagulans* lysate and supernatant treatment did not significantly affect tibia BMD compared to the control and OVX-untreated groups. Recent studies revealed other mechanisms for probiotic effects on bone. *Lactobacillus reuteri* prevented ovariectomy-induced bone loss via changes in bone marrow CD4+ T cells [37]. *Lactobacillus casei* supplementation repressed osteolysis and the pro-inflammatory state of the macrophages [38]. In another work, *Lactobacillus reuteri* 6475 improved bone health by reducing tumor necrosis factor (TNF) levels and decreasing bone resorption. The results showed an increased bone fracture, BMD, BMC, trabecular number and thickness, and falling trabecular space in both vertebral and femoral bones [39]. Parvaneh et al. presented that *Bifidobacterium longum* treatment augmented BMD, but rather than decreasing bone resorption markers, they observed increased bone formation [40].

Postbiotics are defined as extracellular or intracellular substances produced through the metabolic activity of the microorganism in a different phase of growth and could utilize a favorable effect on the host, directly or indirectly [41]. According to the above definition, postbiotics are classified into different classes, including cell-free supernatants, exopolysaccharides, enzymes, cell wall fragments, short-chain fatty acids (SCFA), and bacterial lysates [42]. Postbiotics exhibit pleiotropic activities in the human body. The mechanisms of their health benefits are not clearly defined but might be through immunomodulatory effects, antitumor effects, infection prevention, anti-atherosclerotic effects, and autophagy induction [42].

*Lactobacillus acidophilus* and *Lactobacillus casei* supernatants have anti-inflammatory and antioxidant effects on intestinal epithelial cells, macrophages, and neutrophils by reducing the secretion of the pro-inflammatory tumor necrosis factor α (TNF-α) cytokine and increasing the secretion of the anti-inflammatory cytokine interleukin 10 (IL-10) [43]. Postbiotics originating from *Lactobacillus* include valuable compounds such as organic acids and bacteriocin, enhancing the growth of lactic acid bacteria [44]. *Bacillus coagulans* isolated fractions (supernatant, cell wall fragments) induced anti-inflammatory cytokine production and promote T helper (Th)2-dependent immune responses [45].

Quach et al. reported that cell culture supernatant (CCS) fraction from *L. reuteri* 6475 (< 3 kDa) suppressed the differentiation of monocyte/macrophage cell line into osteoclasts [46]. In another study, VPP peptide from *Lactobacillus helveticus* LBK-16H, because of its low bioavailability, did not display preventive activity against ovariectomy-induced bone loss [47]. Rahman et al. exhibited that conjugated linoleic acid inhibits osteoclastogenesis by modulating RANKL signaling [48]. Chen et al. revealed that the supernatant of *Lactobacillus acidophilus* and butanoic acids stimulated the proliferation, differentiation, and maturity of osteoblasts MC3T3-E1 cells was, increased the activity of alkaline phosphatase, elevated concentration of osteocalcin, and the expression of RUNX2, WNT2 and CTNNB1 [49].

Butyrate (an SCFA) induces the differentiation of regulatory T cells (Tregs) in the intestine [50]. Reports highlight the bone-regulating capacities of Treg cells, describing mechanisms where Treg cells blunt bone resorption, stimulate bone formation by promoting the differentiation of osteoblasts, and are pivotal for parathyroid hormone (PTH)-stimulated bone formation [51]. Tyagi et al. reported that oral delivery of *Lactobacillus gasseri* LGG or butyrate to eugonadal young mice increased trabecular bone volume due to stimulation of bone formation [51].

Postbiotic effectiveness is similar to probiotics, and given that postbiotics do not contain live cells, the risks and side effects associated with their intake are minimal compared to probiotics [42]. Postbiotic do not need colonization and could increase the potency of active microorganisms, keep the microorganisms viable and stable in the product at a high dose, improve shelf-life, and simplify packaging and transport [27]. Postbiotics can also be used in situations where it is harder to control and maintain production and storage conditions, such as in developing countries [24]. The postbiotics used in the current study were originated from five native probiotic strains (*Lactobacillus acidophilus*, *Lactobacillus reuteri*, *Lactobacillus casei*, *Bifidobacterium longum*, and *Bacillus coagulans*). In most cases, the current study found that postbiotics revealed similar capacities in ameliorating ovariectomy-induced bone loss as much as a probiotic live-cell, which was explored in our earlier study. The results suggest that postbiotic could be used as a substitute for probiotics in preventing bone loss result from estrogen deficiency, but further studies needed to be done to confirm the present study outcomes. Collectively, the data from the current study suggest that the effects of postbiotics on biochemical and bone parameters may depend on the type of individual species that postbiotics originated from, duration of treatment, the bone compartment examined, and the estrogen deficiency model used. More studies need to be done to explore the optimal administrative dose and duration of the specific postbiotics in protecting ovariectomy-induced bone loss in further animal and clinical investigations. Furthermore, identifying and characterizing the intracellular and extracellular bioactive molecule(s) produced by bacteria that target bone formation and resorption and their exact mechanisms could help determine the substances that can potentially be used for treating post-menopausal osteoporosis.

The strength of our study is that here, for the first time, we compared 12 different postbiotic treatments obtained from common probiotic strains on ovariectomy-induced bone loss. We showed the strain-specific effects of postbiotics and their specific impacts on various bone compartments in the present work. As the limitations, in-depth mechanism of postbiotics effects on ameliorating ovariectomy-induced bone loss was not investigated. Characterization of postbiotics could be useful in finding the most effective compounds with bone-sparing effects. Further, in vivo studies and clinical trials are recommended to be conducted to discover the vast aspects of postbiotics therapy on ameliorating bone loss.

## Conclusion

In the present study, we concluded that among the tested postbiotics, *Bacillus coagulans* derive-postbiotics displayed the best effects in ameliorating bone loss in various bone sites (area, BMC, and BMD) resulted from ovariectomy. Together, the data from the present study revealed that postbiotics similar to probiotics could ameliorate bone loss resulted from estrogen deficiency. Also, the effects of postbiotics on different bone sites are strain-dependent.

## Data Availability

The datasets employed and/or analyzed along the course of the experiment are available from the corresponding author on reasonable request.
